# Organic acids, sugars, vitamin C content and some pomological characteristics of eleven hawthorn species (*Crataegus* spp.) from Turkey

**DOI:** 10.1186/0717-6287-47-21

**Published:** 2014-05-30

**Authors:** Muttalip Gundogdu, Koray Ozrenk, Sezai Ercisli, Tuncay Kan, Ossama Kodad, Attila Hegedus

**Affiliations:** Department of Agriculture Biotechnology, Agriculture Faculty, Yuzuncu Yıl University, Van, Turkey; Department of Horticulture, Agriculture Faculty, Siirt University, Siirt, Turkey; Department of Horticulture, Agriculture Faculty, Ataturk University, Erzurum, Turkey; Department of Horticulture, Agriculture Faculty, Inonu University, Malatya, Turkey; Departement of Pomology, National School of Agriculture, Meknes, Morocco; Faculty of Horticultural Science, Corvinus University of Budapest, Budapest, Hungary

**Keywords:** Crataegus, Diversity, Fruit characteristics, Organic acids

## Abstract

**Background:**

The Hawthorn (*Crateagus* sp.) mostly occurs around the temperate region of the world with a high number of species, producing a fruit with numerous beneficial effects for human health. The aim of the study was to determine organic acid and sugar contents in the fruit of a number of hawthorn species grown in Erzincan province of Turkey.

**Results:**

Citric acid was the predominant organic acid in all hawthorn species and *C. pseudoheterophylla* had the highest citric acid content (23.688 g/100 g). There were not statistically significant differences among hawthorn species (except *C. atrosanguinea* Pojark) in terms of fumaric acid content. *C.pontica* C.Koch had a higher content of vitamin C (9.418 mg/100 g) compared to other species. Fructose was the predominant sugar component in all species and *C. monogyna subsp. monogyna* Joiq had the highest fructose content (18.378 g/100 g).

**Conclusions:**

The high fruit quality of the studied species indicates the importance of this fruit in human nutrition as a natural source. The study revealed that there were differences in terms of fruit characteristics among hawthorn species and thus better quality hawthorn genotypes can be selected within the species. Hence, this study is considered to be a valuable reference for forthcoming studies. The high fruit quality of the studied species indicates the importance of this fruit in human nutrition as a natural source.

## Background

There has been a keen interest to wild edible fruits including hawthorn that have not been cultivated in common orchards. Their fruits have numerous beneficial effects for human health [1]. The consequence of this neglect is that wild edible fruit species used in developing countries are poorly known. Hawthorn (*Crateagus* sp.) mostly occurs around the temperate region of the world with a high number of species but the main problem is recognizing species [2].

Turkey is a significant genetic center of hawthorn (*Crataegus* spp.) and wild growing hawthorn species has a wide distribution. Hawthorn species are members of the family *Rosaceae*, subfamily *Maloideae*, tribe *Crataegeae* and genus *Crataegus*. Turkey is one of the motherlands of the species that is widely distributed in North Europe, temperate regions of Asia, Africa and North America [2]. There are 165–200 hawthorn species worldwide and about 17 hawthorn species can be found in Turkey [2]. Diverse *Crataegus* species grow in different regions of the world [3]. Such worldwide rich species diversity is attributed to birds and other animals and they disperse its seeds. Hawthorns are deciduous and thorny trees or bushes which have plentiful and conspicuous flowers in bunches in spring; have yellowish, orange or reddish fruits in autumn; have lobbed leaves which also turn similar colors as fruits in fall [4, 5].

Organic acid contents of fruits vary according to species. In particular, sugar–acid balance and contents are the primary determinants of taste attributes of fruit. Organic acids in fruits and vegetables mostly form compounds such as salt, ester and glycoside [6]. Since the acids in fruits are immediately oxidized, they do not have harmful effects on the body. As their salts are alkaline forming elements, they are highly important in human nutrition [6]. Due to their beneficial effects in preventing cardiovascular diseases, hawthorn species have recently become important in nutrition and nutraceuticals [7]. Fruit acids that enable digestion of nutrients and stimulate blood circulation are among the quality parameters for hawthorn fruits.

Hawthorn fruits and leaves have abundant content of flavonoids and procyanidins which have antioxidant, free radical scavenging, anti-inflammatory, vasorelaxing and hypolipidemic properties [8–11]. Flowers and fruits are used in pharmaceutics for medicinal purposes. They help to lower blood tension and strengthen the heart. Hawthorn berries contain glycosides which boost heart health. They are used to treat heart beat irregularities due to aging or neural disorders. They are also good for atherosclerosis, insomnia, seasonal mood swings and nervous system disorders. The herb is non-poisonous and can be used safely for long-term treatments [12]. The berries have laxative and diuretic properties [13]. They contain triterpene acids, choline, acetylcholine, trimethylamine, caffeic and ascorbic acids, aminoacids, adenine and adenosine [12]. Vitamins B1, B2, B6 and C as well as 17 aminoacids (about 3.1%) are determined in hawthorn berries [3].

Recent studies have demonstrated the important role of hawthorn fruits in human health and nutrition. Accordingly, hawthorn fruits are becoming more popular and their consumption has been increasing recently. The hawthorn species used in this study are abundantly grown in Turkey. The study aimed to determine organic acid, sugar, vitamin C content and some pomological characteristics of 11 wild hawthorn species grown in Erzincan region, Turkey. These parameters are important for the determination of quality of hawthorn fruits. The study is important for exploring organic acid and sugar contents in hawthorn species on which only limited literature is available.

## Results and discussions

There were statistically significant differences among species in terms of morphological (fruit weight, fruit dimensions, seed number, seed weight, fruit stalk length and width) and biochemical (SSC, soluble solid content, pH, acidity (Table 1). *C. pseudoheterophylla* Pojark. had the highest fruit weight (3.48 g). In terms of SSC, *C. monogyna subsp. azarella* Jocq. had the highest SSC content (20.00%) while *C. szovitsii* Pojark. had the lowest amount (2.35%) (Table 1). The highest acidity levels were identified respectively in *C. monogyna subsp.azarella* Jocq. (5.99%) and *C. aroni var. aroni* (L) Bosc.ex DC. (2.40%) (Table 1). The fruit weights of hawthorn genotypes were reported previously between 0.81-2.14 g [13]. In some *Crataegus* (Chinese hawthorn) genotypes, the fruit length was reported to be range between 1.50-2.90 mm, the fruit width between 1.60-3.60 mm and the fruit weights between 3.4-16 g [3]. In the literature, the acidity levels of *C. pinnatifida* and *C. altaica* were given as 1.5-4.5% and 0.56%, respectively [14]. Research has demonstrated that morphological and biochemical characteristics of fruits are affected by genetic factors, climatic factors, climate and soil structure. While some findings of this study are in agreement with those of other researchers, some findings are in discord. This is attributed to the variability of hawthorn species used in the studies as well as other environmental factors.

**Table 1:** **Pomological characteristics and Soluble Solid Content (SSC)**, **pH and acidity of hawthorn species** (**fresh weight basis**)

Species	Fruit weight (g)	Fruit height (mm)	Fruit width (mm)	Seed number	Seed weight (g)	Fruit stalk length (mm)	Fruit stalk thickness (mm)	SCC (%)	pH	Acidity (%)
*C. pontica*	2.29 c*	13.13 bcd	12.15 c	1.00 e	0.24 f	7.21 b	0.87 cde	17.51 b	4.58 b	0.88 c
*C. atrosanguinea*.	1.49 d	13.51 bc	13.36 c	4.00 b	0.45 de	0.83 d	0.89 b-e	11.61 c	4.29 b	1.65 b
*C. meyeri*	0.58 e	10.86 e	10.38 d	1.00 e	0.13 g	13.60 a	0.36 de	3.05 e	5.06 ab	0.22 f
*C. pseudoheterophylla*	3.48 a	1.67 f	2.21 e	4.00 b	0.68 ab	1.71 d	0.10 e	7.75 d	4.22 b	0.46 e
*C. orientalis var. orientalis*	3.20 ab	15.56 a	17.68 a	4.00 b	0.75 a	4.14 c	1.63 ab	11.85 c	4.26 b	1.78 b
*C. monogyna subsp. azarella*	2.93 b	14.75 ab	14.80 b	2.00 d	0.54 cd	1.57 d	1.90 a	20.00 a	5.99 a	0.41 e
*C. monogyna subsp. monogyna*	1.35 d	13.12 bcd	12.55 c	1.00 e	0.29 f	7.63 b	0.82 cde	18.25 b	4.73 ab	0.82 c
*C. curvisepala*	0.62 e	11.20 de	10.02 d	1.00 e	0.26 f	13.32 a	0.67 cde	3.35 e	5.22 ab	0.65 d
*C. szovitsii*	1.25 d	12.54 cde	12.69 c	3.00 c	0.27 f	8.14 b	1.37 abc	2.35 e	4.50 b	0.31 ef
*Crataegus pentagyna*	1.60 d	1.29 f	1.44 e	5.00 5	0.61 bc	1.58 d	0.20 e	12.02 c	4.36 b	0.45 e
*C. aroni var. aroni*	3.00 b	15.72 a	17.67 a	4.00 b	0.43 e	7.18 b	1.05 bcd	11.50 c	4.61 b	2.40 a

*There were significant (P < 0.01) differences among the different letters in the same columns.

Organic acids are known to affect particularly taste formation and many physiological processes. Oxalic acid, citric acid, tartaric acid, succinic acid, fumaric acid and malic acid contents in fruits of 11 hawthorn fruit species were examined in this study (Table 2).

**Table 2:** **Fruit organic acid contents of hawthorn species** (**g**/**100 g fresh weight basis**)

Species	Oxalic acid	Citric acid	Tartaric acid	Succinic acid	Fumaric acid	Malic acid
*C. pontica*	1.453 ± 0.024 f*	3.128 ± 0.007 e	1.205 ± 0.040 c	2.174 ± 0.197 b	0.155 ± 0.001 b	1.045 ± 0.016 k
*C. atrosanguinea*.	12.419 ± 0.054 a	19.218 ± 0.027b	0.605 ± 0.026 d	1.082 ± 0.148 i	0.235 ± 0.025 a	2.246 ± 0.038 c
*C. meyeri*	1.513 ± 0.014 ef	2.548 ± 0.092 f	1.668 ± 0.101 b	1.392 ± 0.073 g	0.019 ± 0.000 b	1.518 ± 0.021 g
*C. pseudoheterophylla*	2.461 ± 0.036 cd	23.688 ± 0.041 a	2.217 ± 0.038 a	2.581 ± 0.163 a	0.016 ± 0.000 b	2.671 ± 0.050 a
*C. orientalis var. orientalis*	2.687 ± 0.040 c	2.532 ± 0.056 f	1.579 ± 0.063 b	1.285 ± 0.102 h	0.009 ± 0.000 b	1.347 ± 0.043 i
*C. monogyna subsp.azarella*	3.276 ± 0.147 b	5.665 ± 0.236 d	0.763 ± 0.034 d	1.970 ± 0.225 d	0.011 ± 0.000 b	2.039 ± 0.015 e
*C. monogyna subsp. monogyna*	2.650 ± 0.167 c	1.953 ± 0.010 g	0.780 ± 0.016 d	1.080 ± 0.057 i	0.027 ± 0.001 b	1.721 ± 0.024 f
*C. curvisepala*	2.335 ± 0.036 d	3.245 ± 0.72 e	1.474 ± 0.087 b	1.580 ± 0.054 e	0.013 ± 0.001 b	1.167 ± 0.014 j
*C. szovitsii*	0.537 ± 0.009 g	3.378 ± 0.078 e	1.094 ± 0.085 c	1.556 ± 0.113 ef	0.011 ± 0.000 b	1.472 ± 0.055 h
*Crataegus pentagyna*	1.741 ± 0.042 e	5.641 ± 0.170 d	1.145 ± 0.102 c	2.133 ± 0.113 c	0.015 ± 0.000 b	2.542 ± 0.042 b
*C. aroni var. aroni*	1.470 ± 0.021 f	10.696 ± 0.055 c	0.735 ± 0.025 d	1.530 ± 0.056 f	0.025 ± 0.001 b	2.088 ± 0.028 d

*There were significant (P < 0.01) differences among the different letters in the same columns.

There were statistically significant differences among species in terms of organic acid contents (except fumaric acid) (p < 0.05). *C. pseudoheterophylla* Pojark. had the highest citric acid content (23.688 g/100 g) while *C. monogyna subsp. monogyna* Joiq. had the lowest (1.953 g/100 g) citric acid content. The highest malic acid content was measured in *C. pseudoheterophlla* Pojark. (2.671 g/100 g) and the lowest malic acid content was determined in *C.pontica* C.Koch. (1.045 g/100 g) (Table 2). *C. atrosanguinea* Pojark. had the highest oxalic acid content (12.419 g/100 g). Liu et al. [11] conducted a study on *C. pinnatifida* var. *major* fruits grown in China and reported malic acid content as 0.72 g/100 g and quinic acid content as 1.87 g/100 g. The same researchers also studied on *C. brettschneideri* hawthorn species and identified malic acid content as 0.68 g/100 g, citric acid content as 4.56 g/100 g and quinic acid content as 0.61 g/100 g. In another study, tartaric acid and succinic acid contents were explored in the hawthorn species of *C. pinnatifida var*. major, *C. scabrifolia*, *C. kansuensis*, *C. hupehensis*, and *C. cuneata*[15]. While some of the values determined in this study were close to the values reported in other studies, some values were found to be higher and some were found to be lower. These variations can be attributed to different genetic characteristics of study materials as well as climatic conditions and other environmental factors.

The glucose, fructose, saccharose and vitamin C contents of hawthorn species were also investigated. There were statistically significant differences among species in terms of sugar and vitamin C contents (p < 0.05) (Table 3). *C. monogyna subsp. monogyna* Joiq. had the highest glucose (13.893 g/100 g) and fructose (18.378 g/100 g) contents. The lowest glucose content (6.672 g/100 g) was determined in *C. szovitsii* Pojark and the lowest fructose content (8.527 g/100 g) was detected in *C.pontica* C.Koch. *Crataegus pentagyna* and *C. monogyna subsp. monogyna* had the highest (1.564 g/100 g) and lowest (0.970 g/100 g) saccharose contents, respectively (Table 3).

**Table 3:** **Sugars and vitamin C contents of examined hawthorn species** (**fresh weight basis**)

Species	Glucose (g/100 g)	Fructose (g/100 g)	Saccharose (g/100 g)	Vitamin C (mg/100 g)
*C. pontica*	7.131 ± 0.102 h*	8.527 ± 0.051 e	1.205 ± 0.045 cd	9.418 ± 0.014 a
*C. atrosanguinea*.	9.907 ± 0.003 d	16.619 ± 0.076 b	1.305 ± 0.145 bc	2.805 ± 0.126 g
*C. meyeri*	9.416 ± 0.051 ef	9.591 ± 0.118 e	1.395 ± 0.074 abc	2.780 ± 0.184 g
*C. pseudoheterophylla*	12.513 ± 0.057 b	16.861 ± 0.100 b	1.269 ± 0.076 c	3.738 ± 0.242 e
*C. orientalis var. orientalis*	11.821 ± 0.143 c	16.633 ± 0.156 b	1.517 ± 0.050 ab	5.390 ± 0.102 c
*C. monogyna subsp.azarella*	7.527 ± 0.166 g	11.386 ± 0.119 c	0.999 ± 0.035 de	8.091 ± 0.087 b
*C. monogyna subsp. monogyna*	13.893 ± 0.021 a	18.378 ± 0.202 a	0.970 ± 0.005 e	4.301 ± 0.121 d
*C. curvisepala*	9.194 ± 0.073 f	11.536 ± 0.064 c	1.173 ± 0.003 cde	4.182 ± 0.006 d
*C. szovitsii*	6.672 ± 0.193 i	10.257 ± 0.163 d	0.979 ± 0.025 de	3.260 ± 0.016 f
*Crataegus pentagyna*	9.536 ± 0.079 e	11.404 ± 0.250 c	1.564 ± 0.105 a	4.439 ± 0.064 d
*C. aroni var. aroni*	7.074 ± 0.072 h	10.593 ± 0.132 d	0.954 ± 0.008 e	1.555 ± 0.091 h

*There were significant (P < 0.01) differences among the different letters in the same columns.

In terms of vitamin C content, *C.pontica* C.Koch. had the highest (9.418 mg/100 g) and *C. aroni var. aroni* (L) Bosc.ex DC. had the lowest (1.555 mg/100 g) contents (Table 3). Fructose contents were generally found to be higher than glucose and saccharose contents in this study. In a study on *Crataegus pinnatifida* var. *major* grown in China, fructose content was determined as 13.40 g/100 g, glucose content as 11.67 g/100 g and saccharose content as 5.61 g/100 g [11]. Peaches and apricots have almost equal amounts of glucose, fructose and saccharose. In other hard seeded fruits and berry fruits, glucose and fructose contents are more predominant. Monosaccharides are important since they produce hydroxymethylfurfural by food processing technology with the effects of temperature and acid [16].

The hawthorn species (*C. szovitsii* Pojark., *C.pontica* C.Koch., *C. aroni var. aroni* (L) Bosc.ex DC., *C. atrosanguinea* Pojark., *C. meyeri* Pojark., *C. curvisepala* Lindman, *C. monogyna subsp. monogyna* Joiq., *C. pseudoheterophlla* Pojark., *C. orientalis var. orientalis* Pallas ex Bieb., *C. monogyna subsp.azarella* Jocq., *Crataegus pentagyna*.) that are particularly widespread in the Eastern Anatolia Region of Turkey were examined in this study. In terms of organic acid distribution, citric acid content was identified to be higher than the contents of other organic acids and citric acid was found to be the predominant organic acid in all hawthorn species. Compared to the findings of Liu et al. [11], similar glucose and fructose contents were identified in this study while saccharose contents were lower. Liu et al. [11] demonstrated that while some hawthorn fruit species contained saccharose, some species lacked this compound. In this study conducted in Erzincan region, all examined hawthorn fruit species were found to contain saccharose. Glucose and fructose are the predominant sugar compounds in all fruits and vegetables. Saccharose and mannose may also exist in lower amounts. Recent studies on hawthorn have demonstrated that hawthorn berries are good for boosting heart health, regulating heart beat irregularities, alleviating spasms and lowering blood pressure in addition to their laxative, diuretic and antitumoural properties [13]. These beneficial health effects increased the awareness about the importance of this fruit in nutrition.

## Conclusions

In the literature there are a limited number of studies on morphological and biochemical contents in hawthorn fruits. Particularly the study included a number of hawthorn species and hence this study is considered to be a valuable reference for forthcoming studies on morphological and biochemical characteristics of the species to find the most favorable one to introduce it into for bring them commercial production. The results suggested its high potential of health benefits. However, more detailed biological and pharmacological studies are needed for the demonstration and clarification of health benefits of hawthorn fruits.

## Methods

### Plant materials

Homogeneous fruits samples were collected at the harvest time of the determined *C. szovitsii* Pojark., *C.pontica* C.Koch., *C. aroni var. aroni* (L) Bosc.ex DC., *C. atrosanguinea* Pojark., *C. meyeri* Pojark., *C. curvisepala* Lindman, *C. monogyna subsp. monogyna* Joiq., *C. pseudoheterophlla* Pojark., *C. orientalis var. orientalis* Pallas ex Bieb., *C. monogyna sııbsp.azarella* Jocq., *Crataegus pentagyna* fruits grown in Erzincan province. Altitude of Erzincan is 1214 m. Fruit samples were collected from towns (Otlukbeli, Kemaliye, Çayırlı, İliç) connected with Erzincan. Erzincan has a temperate climate with annual average temperature of 10.7°C. The average annual rainfall in the province is 344 mm/m^2^.

### Morphological and biochemical characteristics of fruits

Thirty matured hawthorne fruits were selected based on morphological characteristics for fruit analyses. Desirable morphological and biochemical characteristics such as fruit weight (g), fruit length (mm), fruit diameter (mm), seed weight (g), seed number, fruit stalk length (mm), fruit stalk thickness (mm), fruit juice pH, soluble solid content (SSC,%) and acidity (%) were determined according to Cemeroglu [17]. Fruit samples from each species were maintained at -20°C before organic acids, sugars and vitamin C analysis.

### Extraction and determination of organic acids

In the present study, chemicals with analytical purity were used. Organic acid standards (citric acid, tartaric acid, oxalic acid, malic acid, succinic acid and fumaric acid), sugar standards (glucose, fructose, and sucrose), and vitamin C standards (l-ascorbic acid) were obtained from Sigma–Aldrich (St. Louis, MO, USA). The other chemicals were obtained from Merck (Darmstadt, Germany).

For organic acid extraction, the method by Bevilacqua and Califano [18] was modified. About 200 g of samples was fragmented and 10 g from each sample was delivered to centrifuge tubes. The 10 ml of 0.009 N H_2_SO_4_ was added to the samples and the samples were homogenized with Heidolph Silent Crusher M, Germany. Then, the samples were mixed for an hour with a shaker (Heidolph Unimax 1010, Germany) and centrifuged at 14.000 × rpm for 15 min. The supernatants were passed through coarse filter paper, then twice through a 0.45 mm membrane filter (Millipore Millex-HV Hydrophilic PVDF, Millipore, USA), and last in a SEP-PAK C18 cartridge. The concentration of organic acids was determined by HPLC using an Aminex column (HPX-87H, 300 mm × 7.8 mm, Bio-Rad) fitted on an Agilent 1100 series HPLC G 1322 A, Germany) [18]. Organic acids were detected at 214 and 280 nm wavelengths. The mobile phase, 0.009 N H_2_SO_4_ was passed through a 0.45 μm filter membrane.

### Extraction and determination of sugars

The samples were prepared according to the method described by Melgarejo et al. [19] with minor modifications. Briefly, the samples of 10 g fruit were centrifuged at 12.000 rpm for 2 min at 4°C. Then the supernatant was filtrated with SEP-PAK C18 cartridges and transferred into a vial and used for analysis. Analysis of sugars was performed by HPLC (isocratic program) equipped with a μbondapak-NH_2_ column and a refractive index (RI) detector using 85% acetonitrile as a mobile phase. The calculation of concentrations was based on standards prepared in the laboratory.

### Extraction and determination of ascorbic acid (vitamin C)

Ascorbic acid content was determined following the modified HPLC (isocratic program) (Agilent 1100 series HPLC G 1322 A, Germany) analytical procedure outlined by Cemeroglu [20]. The 10 g of sample was delivered to a 50 ml volumetric flask including 10 ml 6% (w/v) metaphosphoric acid (Sigma, M6285, 33.5%). The sample was then homogenized at 24.000 rpm for 15 s, and centrifuged at 14,000 rpm for 10 min at 1°C. 5 ml of the supernatant was filtered through 0.45 μm PTFE syringe filters (Phenomenex, UK) and placed in an amber colored vial (AIM, Screw vial, SV-15A). Quantification of ascorbic acid was made by an external standard method using an l-ascorbic acid standard (Sigma A5960). Samples were separated on a Luna C18 column (250 mm × 4.60 mm, 5 μm from Phenomenex) at 25°C by an HPLC. The mobile phase was 25 mM KH_2_PO_4_ (adjusted to pH 2.2 with phosphoric acid) with a flow rate of 1 ml/min. L-ascorbic acid was detected at 254 nm.

### Statistical analysis

Experimental data were evaluated using analysis of variance (ANOVA) and significant differences among the means of three replicates (*p* < 0.05) were determined by Duncan’s multiple range tests, using the “SPSS 10.0 for Windows”.

## References

[1] Tadic MV, Dobric S, Markovic MG, Dordjevic MS, Arsic AI (2008). Anti-inflammatory, gastroprotective, free-radical-scavenging, and antimicrobial activities of hawthorn berries ethanol extract. J Agric Food Chem.

[2] Ercisli S (2004). A short review of the fruit germplasm resources of Turkey. Gen Res Crop Evol.

[3] Guo TJ, Jiao PJ (1995). Hawthorn (*Crataegus*) resources in China. HortSci.

[4] Yilmaz KU, Yanar M, Ercisli S, Sahiner H, Taskin T, Zengin Y (2010). Genetic relationships among some hawthorn (*Crataegus* spp.) species and genotypes. Biochem Genetics.

[5] Caliskan O, Kazim G, Serce S, Toplu C, Kamiloglu O, Sengul M, Ercisli S (2012). Phytochemical characterization of several howthorn (*Crataegus* spp) species sampled from the Eastern Mediterranean region of Turkey. Pharmacognosy Maga.

[6] Savran HS (1999). Nar Suyunda Organik Ait Dağılımı (yüksek lisans tezi).

[7] Chang W, Dao J, Shao Z (2005). Hawthorn: potential roles in cardiovascular disease. Amer J Chinese Med.

[8] Quettier-Deleu C, Voiselle G, Fruchart JC, Duriez P, Teissier E, Bailleul F, Vasseur J, Trotin F (2003). Hawthorn extracts inhibit LDL oxidation. Pharmazie.

[9] Cui T, Nakamura K, Tian S, Kayahara S, Tian Y (2006). Polyphenolic content and physiological activities of Chinese hawthorn extracts. Biosci Biotech Biochem.

[10] Bernatoniene J, Masteikova R, Majiene D, Savickas A, Kevelaitis E, Bernatoniene R, Dvorackova K, Civinskiene G, Lekas R, Vitkevicius K, Peciura R (2008). Free radical-scavenging activities of *Crataegus monogyna* extracts. Medicina (Kaunas).

[11] Liu P, Kallio H, Lu D, Zhou C, Ou S, Yang B (2010). Acids, sugars, and sugar alcohols in Chinese hawthorn (*Crataegus* spp.) fruits. J Agric Food Chem.

[12] Tanker N, Koyuncu M, Coskun M (1998). Farmasotik Botanik.

[13] Baytop T (1984). Türkiye’de Bitkiler İle Tedavi (Geçmişte ve Bugün).

[14] Zhao HC, Tian BF (1996). China Fruit-Plant Monograph. Hawthorn Flora.

[15] Gao GY, Feng YX, Qin XQ (1995). Analysis of the chemical constituents of hawthorn fruits and their quality evaluation. Yaoxue Xuebao.

[16] Gundogdu M, Yilmaz H (2012). Organic acid, phenolic profile and antioxidant capacities of pomegranate (*Punica granatum* L.) cultivars and selected genotypes. Sci Hortic.

[17] Cemeroglu B (1992). Meyve ve Sebze İşleme Endüstrisinde Temel Analiz Metodları.

[18] Bevilacqua AE, Califano AN (1989). Determination of organic acids in dairy products by high performance liquid chromatography. J Food Sci.

[19] Melgarejo P, Salazar DM, Artes F (2000). Organic acids and sugars composition of harvested pomegranate fruits. Eur Food Res Tech.

[20] Cemeroglu B (2007). Gıda Analizleri. Gıda Tekn Der Yayı.

